# COVID-19 and investment in applied epidemiology

**DOI:** 10.2471/BLT.22.288687

**Published:** 2022-07-01

**Authors:** Matthew Myers Griffith, Amy Elizabeth Parry, Tambri Housen, Tony Stewart, Martyn D Kirk

**Affiliations:** a National Centre for Epidemiology and Population Health, 62 Mills Road, Australian National University, Acton, ACT 2600 Australia.

Now is the time to invest in applied epidemiology. Applied epidemiology is the intelligence arm of health emergencies: it provides information on a pathogen’s movements and ways to stop it. Most countries had applied epidemiologists when the coronavirus disease 2019 (COVID-19) appeared; yet we needed more applied epidemiologists with better training, and higher government regard. The pandemic caused millions of deaths and social disruptions, so governments must invest in applied epidemiology before the next one.

Applied epidemiologists inform decision-making to improve population health. They help control epidemics by modernizing disease reporting, improving disease indices, informing clinical examination and treatment, advising on laboratory testing and recommending control measures.^1^ Whereas academic epidemiologists value analytic rigour and use high-quality data from controlled circumstances, applied epidemiologists value urgency, speed, practicality, responsiveness and impact; they use real-life data from surveillance and field investigation.^2^

When COVID-19 appeared, too few applied epidemiologists (including field epidemiologists) were working in the field. The World Health Organization (WHO) sets a target of one field epidemiologist per 200 000 population. This target has limitations, such as no standard definitions or job classifications, but helps direct efforts and identify challenges to progress.^3^ A graduate of a field epidemiology training programme has become the proxy for field epidemiologist. Globally, 86 of these programmes existed in 2021,^4^ meaning that many countries could not produce their own graduates. Even where programmes existed, not all their graduates worked in the field. For example, the Jordan training programme had graduated 63 residents as of 2017, making it one of the few countries in the region to meet the WHO target, yet only 39 of them were working as government epidemiologists.^5^ Papua New Guinea had graduated 81 as of 2018 (1.8 per 200 000), but over half of its 89 districts lacked a graduate.^6^

Furthermore, not all graduates acquire competencies for a health emergency such as COVID-19. Global partners and national governments have spread the Epidemic Intelligence Service training approach around the world since the 1970s. In 2003, the United States Centers for Disease Control and Prevention made a standard curriculum to improve consistency across programmes, among other reasons.^7^ In 2019, funders, implementers and stakeholders of these programmes noted that expansion and diversification brought challenges for assuring quality and updating curricula.^8^ A survey of applied epidemiologists in 64 countries shows gaps in data analysis, epidemiology methods and social and communication skills.^9^

Government regard affects the number of competent applied epidemiologists working in the field. Governments can support training programmes and facilitate graduates to work in public health. Yet, in 2019, slow in-country institutionalization of training programmes and a lack of career paths for graduates surfaced as key challenges to epidemiology capacity.^8^ Without funding, programmes seek external support, which comes with demands that may not meet national needs. Without career paths, recruits do not join and graduates go to private institutes or abroad.

Insufficient numbers, inadequate training and low government regard reduce applied epidemiology’s capacity to investigate epidemics and inform decisions to control them. Governments that seek informed, timely decisions to prevent epidemics from becoming pandemics must invest in applied epidemiology. We urge governments not to create a training programme where one already exists. We caution against investing in data technologies while the people who collect, report, interpret and strengthen data lack the numbers, training and recognition to do it. Instead, we recommend five actions. 

First, establish pathways for applied epidemiologists to work in government public health. Career paths attract talent and convert investment in training into investment in the public’s health. Governments should classify epidemiologists in human resources schemes and clarify pathways for promotion. 

Second, institutionalize applied epidemiology training programmes. National funding avoids incompatible external demands and political cycles. Governments should secure line-item budgets for their training programmes. 

Third, inform epidemic and pandemic decision-making with applied epidemiology. Applied epidemiologists interpret epidemic information and inform decisions. Governments should appoint them to advisory roles, involve decision-makers in their training programme activities and facilitate trainees to investigate local outbreaks. 

Fourth, establish training programmes where they do not yet exist. At minimum, every 200 000 people should have a trained, competent field or applied epidemiologist to detect and investigate epidemics. Governments should work with partners to build programmes that meet local needs. 

Fifth, ensure training approaches fit the purpose. Investment must produce a competent workforce that serves populations and informs decision-makers. Governments should review training competencies – considering the lessons of COVID-19 – and engage learning science professionals to develop training that matches trainees’ learning needs and styles.

WHO advocates that we have the duty to do things differently, to invest to create fairer and more resilient health systems.^10^ Applied epidemiology is an intelligent investment. 
